# Study on the insecticidal activity of entomopathogenic fungi for the control of the fruit fly (*Anastrepha obliqua*), the main pest in mango crop in Colombia

**DOI:** 10.1007/s00203-023-03405-2

**Published:** 2023-02-06

**Authors:** María Denis Lozano-Tovar, Karen Lorena Ballestas Álvarez, Luis Andrés Sandoval-Lozano, Gloria Milena Palma Mendez, Gloria Patricia Barrera-Cubillos

**Affiliations:** 1 Corporación Colombiana de Investigación Agropecuaria (AGROSAVIA). Centro de Investigación Nataima, Km 9, Espinal-Ibagué, Tolima, Colombia; 2 Universidad del Tolima, Cl. 42 #1b-1, Ibagué, Tolima, Colombia; 3 Instituto Colombiano Agropecuario (ICA) Cra, 1a #42-63, Ibagué, Tolima, Colombia; 4 Corporación Colombiana de Investigación Agropecuaria (AGROSAVIA). Centro de Investigación Tibaitata, Km14 Mosquera-Bogotá, Tolima, Colombia

**Keywords:** Secondary metabolites, Entomopathogenic fungus, Destruxins, Biological control, *Mangifera indica*

## Abstract

The aim of this study was to evaluate and select entomopathogenic fungi that produces insecticidal compounds for the control of adults of *Anastrepha obliqua* Macquart (Diptera: tephritidae) that are the main pest of mango (*Mangifera indica* L. Bark) in Colombia. Nine entomopathogenic fungi isolates were evaluated, five belonging to the genus *Metarhizium* and four belonging to the genus *Beauveria.* One strain of the species *Metarhizium robertsii* with insecticidal activity was selected. By column fractionation, an active fraction was obtained, which caused mortalities higher than 90% after 48 h of exposure. Through HPLC it was determined that the active fraction is composed of more than 22 metabolites. Identification of the metabolites by UHPLC MS/MS revealed the presence of destruxin in E, D, A and B groups (destruxin E-diol, destruxin D, destruxin D1, destruxin D2, destruxin A2, destruxin A, destruxin A3, dihydrodestruxin A, desmB, destruxin B2, destruxin B and destruxin B1). The evaluation of the insecticidal capacity of the organic fractions obtained by HPLC indicated that the extract obtained from the isolate *M. robertsii* had a compound with high activity on adults of *A. obliqua* (destruxin A) causing massive mortality of up to 100%, after 48 h of the treatment administration. Furthermore, two other compounds with medium activity were found (destruxin A2 and destruxin B), showing mortalities between 60.0 and 81.3%, respectively. The extract of the isolate MT008 of *M. robertsii* showed higher insecticidal activity and a potential source for the control of *A. obliqua*

## Introduction

Fruit flies (Diptera: Tephritidae) are an important pest for fruit-producing countries due to their high incidence and severity which conduces to quarantine restrictions and economic losses (Ekesi et al. 2016; Pérez et al. 2008). In this regard, *A. obliqua* (the West Indian fruit fly) is the main pest in mango and guava (*Psidium guajava* L.) that it also infests species of the botanical families Anacardiaceae, Annonaceae, Bignoniaceae, Fabaceae, Myrtaceae and Rosaceae (Oroño et al. 2006). *A. obliqua* has an extremely wide distribution with records ranging from the south of the USA to the northern region of Argentina, extending from Mexico to Panama, Colombia, Venezuela, Ecuador and Brazil with farther distribution throughout the Caribbean in Jamaica and Trinidad (Norrbom and Korytkowski 2007). In regard to Colombia, the genus *Anastrepha* includes 47 species; five of them have quarantine importance: *A. fraterculus* (Wiedemann), *A. grandis* (Macquart), *A. obliqua* (Macquart), *A. serpentina* (Wiedemann) and *A. striata* (chinner) (Canal 2010). The distribution of this genus in Colombia ranges from the sea level to 2000 m a.s.l. (Martínez 2007); it is estimated that more than 40% of the fruit production in Colombia is already affected by several species of the Tephritidae family. According to information provided by the Instituto Colombiano Agropecuario [Colombian Institute of Agriculture] (ICA 2015), “Colombia, does not have areas free of fruit flies; however, there are areas with low prevalence of this pest. Nonetheless, this limits the domestic market as well as exports” (Giraldo et al. 2015). Furthermore, the excessive use of chemical insecticides for fruit fly control, for more than 50 years, has caused important effects on ecosystems as well as on human health (Haniotakis 2005). The repeated use of a very limited number of active ingredients in these insecticides (organophosphates, carbamates, pyrethroids and spinosads) allow future generations of fruit flies to be resistant to these additives, resulting in a loss of effectiveness (Margaritopoulos et al. 2008; Kakani et al. 2008, 2010).

On the other hand, entomopathogenic fungi are the most predominant controllers for arthropod populations in nature, thus the great potential of these organisms in the regulation of crop pests (Lacey et al. 2015). Extracts obtained from entomopathogenic fungi have shown high insecticidal activity in several studies. For instance, extracts obtained from *Metarhizium* isolates have proved to be toxic for adults of the Mediterranean fruit fly (*Ceratitis capitata* Wiedemann), with mortalities close to 90% (Castillo et al. 2000). Likewise, extracts obtained from isolates of the fungus *Beauveria* spp. and *Metarhizium* spp. produce secondary toxic metabolites that affect adults of *C. capitata* and *Bactrocera olea* (Rossi) with mortalities above 80% after 48 h of application (Yousef et al. 2013; Lozano-Tovar et al. 2015). Other compounds with high molecular weight (soluble proteins) from *Metarhizium brunneum* have shown chronic insecticidal activity after being fed to adults of *C. capitata* (Ortiz-Urquiza et al. 2009). Perhaps destruxins (dtx) are one the most researched secondary metabolites of entomopathogenic fungi. Insect susceptibility to dtxs is variable, but in general, dtxs A and E seem to be more toxic compared to dtxs D and B (Hu et al. 2009; Lozano-Tovar et al. 2015).

Taking this background into account, the aim of this study was to select the entomopathogenic fungi with the insecticidal capacity and identify its bioactive compounds for the generation of biological management alternatives for the control of the fruit fly (*Anastrepha obliqua*), the highest mango pest in Colombia.

## Materials and methods

### Biological material

#### Microorganisms

Nine entomopathogenic fungi isolates supplied by the work collections of Corporación Colombiana de Investigación Agropecuaria AGROSAVIA were used. Five belong to the genus *Metarhizium* (MT005, MT007, MT008, MT009 and MT040), and four belong to the genus *Beauveria* (BV002, BV009, BV012 and BV016) (Table 1).

**Table 1:** Isolates used in this study

Isolate code	Genus	Origen* and host
MT005	*Metarhizium* spp	Melolonthidae larvae. Rionegro (Antioquia)
MT007	*Metarhizium* spp	Nematode.Cajamarca (Tolima)
MT008	*Metarhizium* spp	*Rhammatocerus schistocercoides* Villavicencio (Meta)
MT009	*Metarhizium* spp	*Rhammatocerus schistocercoides*. Puerto Gaitan (Meta)
MT040	*Metarhizium* spp	Melolonthidae larvae. Rionegro (Antioquia)
BV002	*Beauveria* spp	*Premnotrypes vorax*. Savanna of Bogotá (Cundinamarca)
BV009	*Beauveria* spp	Coleoptera. Puerto Gaitan (Meta)
BV012	*Beauveria* spp	Coleoptera. Rionegro (Antioquia)
BV016	*Beauveria* spp	Melolonthidae adults. Cajamarca (Tolima)

*Colombian regions

#### Insects

Adults of *A. obliqua* were obtained from standardized breeds from the Instituto Colombiano Agropecuario (ICA), Regional Office of Ibagué, Tolima, Colombia. Insects were maintained at 25 ± 2 °C and humidity of 65 ± 5% with a photoperiod consisting of 12 h of light and 12 h of darkness. Each breeding room has LG Inverter 9000 BTU air conditioners, air humidifiers (Wunder) with a 1.6-L water tank and a Halux brand digital timer for indoor use where the time for lights on and off is programmed. Humidity and environmental temperature are measured daily (morning and afternoon) with the help of a Fisherbrand Traceable thermo-hygrometer.

### Location

The process of obtaining active compounds and their evaluation were executed in the microbiology laboratory of C.I. Nataima, AGROSAVIA. The research station is located at an altitude of 418 m a.s.l., at 4° 11′ 31.65'' of Latitude N and 74° 57′ 41.49'' of Longitude W in the department of Tolima, municipality of El Espinal, Colombia.

### Selection of microorganisms with insecticidal capacity

#### Production of crude extracts

Isolates were placed in petri dishes of 9 cm diameter containing sabouraud dextrose agar culture medium (Merck KGaA, Germany). Incubation was carried out for 7 days (d) at 28 ± 2 °C. Then the conidia were collected, and a suspension of them was prepared (1 × 10^7^ conidia/ml); then, 2 ml of this suspension were transferred to 250 ml of liquid medium G20P20 (20 g of glucose, Merck KGaA, Germany and 20 g of peptone Merck KGaA, Germany, per liter) contained in flasks of one liter capacity. The liquid culture was incubated for 20 d under the specified conditions. Then the cells were filtered with paper (Whatman No.3) and centrifuged (Heal Force Model Neofuge 23R) at 10,000 g for 20 min, and then, supernatants were preserved at  – 20 °C until their use.

#### Evaluation of crude extracts

The supernatants were evaluated both concentrated and non-concentrated. Supernatants were concentrated through drying using an air current at a temperature of 28 ± 2 °C and then resuspended in a volume 15 times less than the initial. Subsequently supernatants were mixed with hydrolyzed protein and sugar at a 4: 1 ratio, and this was given daily to *A. obliqua* adults in doses of 100 μL of the treatment per repetition and placed in plastic wells of 150 μl.

Adult mortality was recorded periodically after feeding adults with different treatments. Concentrated supernatant mortality was monitored for 66 h; meanwhile, non-concentrated extract mortality was monitored for 5 d. Newly emerged adults were distributed in each experimental unit (ten insects per unit) for a total of 40 insects/treatment. Control treatment consisted of adults fed untreated hydrolyzed protein and sugar 1:1 (mixed with 0.4 gr/ml water).

### Molecular identification of selected isolates

The strains that showed the highest insecticidal activity in this work (MT005 and MT008) were molecularly characterized. The fungal DNA was extracted from conidia using a Zymo research Quick-DNA fungal/bacterial miniprep kit, according to the manufacturer’s protocol.

Characterization was based on the sequencing of the internal transcribed spacer (ITS) region (White et al. 1990), and two additional genes were sequenced: the partial beta-tubulin (Btub) (Tartar et al. 2002) and the partial Elongation Factor EF-1α (Rehner & Buckley 2005). PCRs were carried out in 25 μl, using 5 μl reaction buffer, 6.25 mM dNTPs, 2.5 mM MgCl2, 1 μL of each primer (10 μM), 0.2 μL DNA taq polymerase (Promega) and approximately 100–300 ng of genomic DNA. The general thermal conditions were: 94 °C for 4 min, followed by 35 cycles of 10 s at 92 °C, 20 s at 55 °C and 60 s at 72 °C, and a final extension of 5 min at 72 °C. The products were visualized on a 1.5% agarose gel in TBE buffer, using SYBR® Safe (Thermo Fisher Scientific). The sequences, which were generated by the AGROSAVIA laboratory, were aligned, edited and analyzed by MEGA X (Kumar et al. 2018), and BLASTn was carried out on the GenBank database of the National Centre for Biodiversity Information (NCBI) for identification. Phylogenetic analyses were conducted, using concatenate sequences of ITS, Btub and EF-1α genes using representative sequences of different *Metarhizium* species from the database of the National Center for Biotechnology Information (NCBI) (Table 2) and *Beauveria* species as outgroup. There was a total of 2515 positions in the final dataset for concatenated sequences. The phylogenetic tree was inferred using the neighbor-joining method (Saitou & Nei 1987), and the bootstrap consensus tree was inferred from 1000 replicates (Felsenstein 1985).

**Table 2:** Sequences used in phylogenetic analysis

Specie and strain code	Analyzed region*	GenBank accession number
*Metarhizium anisopliae*	ITS—EF-1α—β-tubulin	PRJNA530366
*Metarhizium acridum* CQMa 102	ITS—EF-1α—β-tubulin	PRJNA245139
*Metarhizium rileyi* RCEF 4871	ITS—EF-1α—β-tubulin	PRJNA72739
*Metarhizium majus* ARSEF297	ITS—EF-1α—β-tubulin	PRJNA302308
*Metarhizium brunneum* ARSEF 3297	ITS—EF-1α—β-tubulin	PRJNA608152
*Metarhizium guizhouense* ARSEF 977	ITS—EF-1α—β-tubulin	PRJNA184755
*Metarhizium robertsii* ARSEF23	ITS—EF-1α—β-tubulin	PRJNA245140
*Beauveria bassiana ARSEF 2860*	ITS—EF-1α—β-tubulin	PRJNA225503
*Beauveria pseudobassiana*	ITS—EF-1α—β-tubulin	PRJNA314175

*White et al. 1990, Tartar et al. 2002, Rehner & Buckley 2005

### Evaluation of the effect of environmental factors on the insecticidal activity of crude extracts obtained from *Metarhizium robertsii*

A variety of environmental factors like temperature and light have been shown to have dramatic effects on the efficacy of entomopathogens against insect pests (Inglis et al. 2001). Mango crops in the country are developed under high temperatures (32 °C) and high solar radiation (700–890 Watt/m^2^), and the effects of these two factors on the insecticidal activity of concentrated supernatants obtained from the selected *Metarhizium robertsii* MT008 and MT005 were evaluated. Concentrated supernatants were exposed to three different temperatures in different times, as follows: room temperature (30 °C ± 2 °C) for 3 h; 50 °C for 3 h; and 120 °C for 20 min. Concentrated supernatants were subjected to 4 h of direct solar radiation (from 10:00 h to 14:00 h) with 784 W/m^2^ irradiance and an average temperature of 32.08 °C (Davis Vantage Pro2 station, C.I. Nataima-Agrosavia). Then the supernatants were exposed to four hours of ultraviolet light in a flow chamber. The concentrated crude extracts remained stored under environmental conditions (30 °C ± 2 °C) in the shade for 0, 24, 48 and 72 h. These extracts were evaluated subsequently on adults of *A. obliqua*. Concentrated supernatants with no exposure to environmental conditions were used as controls.

### Evaluation of insecticidal activity in fractions obtained from MT008 crude extracts

#### Fractionation

The concentrated supernatant from isolate MT008 was fractionated by dialysis, against distilled water (1:20 fungal extract: water) using a dialysis membrane with a molecular weight cutoff of 3500 Da (Spectra/Por®). The process was carried out with constant agitation on magnetic stirrer for 48 h at 4 °C. The two fractions obtained (dialyzed and adialyzed) were evaluated. The dialyzed fraction was subjected to normal phase column fractionation with high-purity grade silica gel, with a pore size of 60 A, and a 70–230 mesh (Sigma-Aldrich) under two elution conditions, 2-propanol-EtOAc (Merck KGaA, Germany) at a ratio of 8:2 and H_2_O-MeOH (Merck KGaA, Germany) at a 7:3 ratio (Lozano-Tovar et al. 2015). Two fractions were obtained: 2-propanol-EtOAc and H_2_O-MeOH. The 2-propanol-EtOAc fraction was subjected to HPLC by a reverse phase chromatography (1260 Infinity System, Agilent Technologies) using a RP-C-18 column (Ascentis, Supelco, of 10 cm × 10 mm × 10 µm). The mobile phase was H_2_O-MeCN: MeCN (in an 80–20: 70 ratio), and it was applied in gradient for 70 min, with a flow of 1.5–2.5 ml/min. The injection volume was 300 μL.

#### Evaluation of fractions

In order to evaluate the effect of the fractions on fruit fly adults, all fractions were mixed with their food (hydrolyzed protein and sucrose in a 4:1 ratio), applying 100 μl of the treatment per repetition. The treatments were carried out in plastic wells of 150 μl of capacity to feed the adults**.** Each treatment was constituted by one of 22 peaks obtained by HPLC fractionation; 0.6 mg of the fractions was resuspended in 300 µl of water. The experimental units were constituted by one-liter containers with tulle fabric as lid. Insects were kept at room temperature (28 ± 2 °C), at a relative humidity of 60% and 12 h photoperiod. Newly emerged adults were distributed in each experimental unit (ten per unit), for a total of 40 insects/treatment.

### Statistical analysis

Treatments were carried out in a randomized block design, with three or four repetitions. Newly emerged adults were distributed in each experimental unit (ten per unit). Data analysis was performed using ANOVA, and the difference in means was established with Tukey’s multiple range test, with an honestly significant difference (HDS 0.5%). Homogeneity of variances and normality of the data were established as well (Lozano-Tovar et al. 2015; Resquín-Romero et al. 2016; Khanday et al. 2018).

### Identification of compounds

The chromatographic profiles of the compounds obtained by HPLC were collected. Each fraction was analyzed by a UHPLC system connected to an Ultimate™ 3000 masses (Thermo Fisher Scientific, USA), equipped with a C18 reverse phase analytical column (2.1 mm × 150 mm, 1.7 µm) (Kinetex Phenomenex, USA) and maintained at 40 °C. The mobile phases used were: water with 0.01% formic acid (A) and methanol with 0.01% formic acid (B). As well, at a flow rate of 0.300 ml/min, the elution gradient was 10–90% of B during 14 min, with an equilibrium of 90% per 2 min, using an injection volume of 20 μl, and samples were resuspended in 1.0 ml of a mobile phase of a mix of A and B (50:50). Subsequently, mass detection was performed in a Q exactive high-resolution mass spectrometer (Thermo Fisher Scientific, USA). The first experiment was performed in full MS mode at a resolution of 70,000, with a 67–1,000 m/z range. The orbitrap was equipped with an electrospray ionization source (ESI), operated in a positive mode (ESI^+^,) with a spray voltage of 3.5 kV, a capillary temperature of 280 ℃ and a heating temperature of 460℃. In a first run, the retention times and the target ions were established, and the ions corresponding to both the hydrogen and the sodium adducts were chosen. This was carried out to establish the molecular ion of the compound. A second run was made, in which the mass spectrometer was set in the mass/mass mode; for this procedure, hydrogen was chosen as adduct using the selective ion mode (SIM); afterward, the higher-energy C-trap dissociation (HCD) was applied, where energies of 10, 30, 50, 70 and 100 arbitrary units were set to generate the fragmentation of the hydrogen adducts. The compounds were tentatively identified by analyzing the data obtained from the orbitrap and using the exact masses and fragmentation patterns of the compounds.

## Results

### Selection of microorganisms due to their insecticidal capacity

**Fig. 1:**
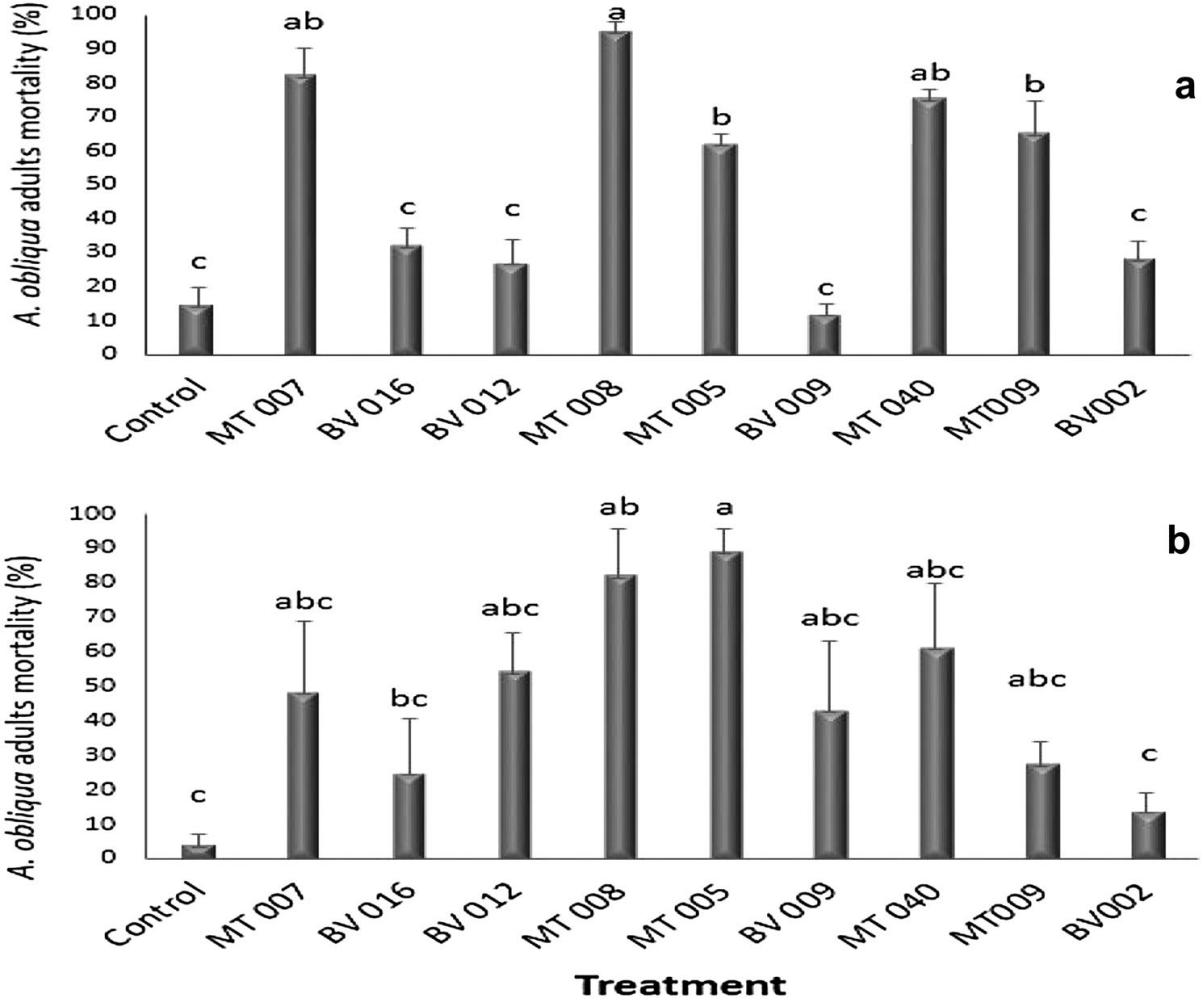
Evaluation of crude extracts obtained from *Metarhizium* spp. and *Beauveria* spp. on adults of *Anastrepha obliqua*. **a** Adult mortality (mortality % ± SE mean), with concentrated supernatants at 66 h after treatment. **b** Adult mortality (mortality % ± SE mean), with non-concentrated supernatants at five days after treatment. Treatments with the same letter do not differ statistically. A multiple range test was run, using Tukey’s honestly significant difference (HDS 0.5%)

Statistical differences between isolates were found (F_9,39_ = 31.74, *P* = 0.0000). To begin with, the concentrated supernatants obtained from the *Metarhizium* spp. isolates showed higher insecticidal activity than those obtained from the *Beauveria* spp. isolates. After 66 h, the treatment was distributed, and it was observed that the average mortality produced by *Metarhizium* spp. was 76.4 ± 3.2%, while the average mortality recorded by the *Beauveria* spp. isolates was 25.2 ± 3.7%. On the other hand, the MT007, MT008 and MT040 isolates are statistically the same and different from the control; however, the MT008 isolate showed the highest mortality (95.5 ± 2.5%) (Fig. 1a). In the same way, statistical differences were found among isolates (F_9,39_ = 4.77*,* *P* = 0.0007), when the non-concentrated extracts were evaluated. The highest mortalities were obtained with the extracts from *Metarhizium* MT005 (89.4 ± 6.1%) and MT008 (82.5 ± 13.1%); these were different compared to the control. A multiple range test was run, using Tukey’s honestly significant difference (HDS 0.5%), that showed mortality values of just 4.5 ± 2.6%, five days after treatment was distributed (Fig. 1b).

### Molecular identification MT005 and MT008 isolates

**Fig. 2:**
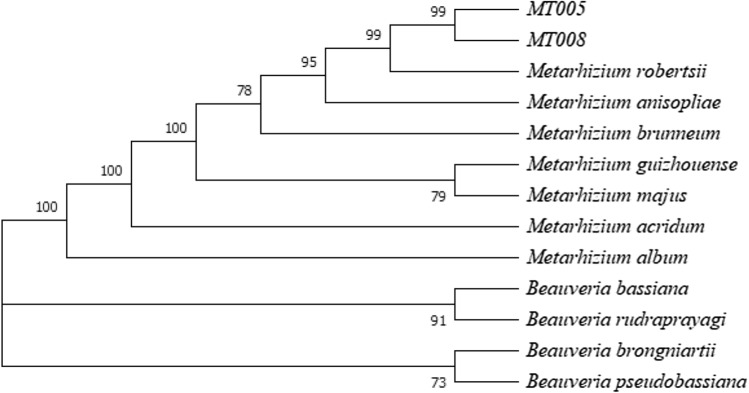
Phylogenetic analysis of concatenated partial sequences of ITS, Btub and EF-1α. Numbers at branches represent bootstrap values. Tree was inferred using the neighbor-joining method

The sequences generated for the MT005 and MT008 corresponded to *Metarhizium robertsii*. No differences were observed between sequences of both isolates. Using the BLASTn function, the closest match of the ITS region was found with the strain of *M. anisopliae* (ARSEF 488/ FJ609303.1: 99%). However, the EF-1α (with the closest match being *M. robertsii* ARSEF 9779/ MK156068.1: 99.85%) and the Btub gene sequences (with the closest match being *M. robertsii* ARSEF 23/ XM_007820079.1: 100%) confirmed the species are *M. robertsii*. The sequences obtained were deposited in the GenBank (ITS: Mt005—MW820167, MT008—MW820168; EF-1α: MT005—MW831678, MT008 – MW831679; Btub: MT005 – MW831676, MT008—MW831677). The neighbor-joining phylogeny of the concatenated sequences of the three regions grouped MT005 and MT008 with *M. robertsii* in the same clade with a high bootstrap value (99%) (Fig. 2). *M. anisopliae* was found in a closely clade with *M. brunneum* and *M. robertsii* with high percentages of bootstrap (78%) (Fig. 2).

### Evaluation of the effect of environmental factors on the insecticidal activity of crude extracts obtained from *Metarhizium robertsii* MT005 and MT008 isolates

**Fig. 3:**
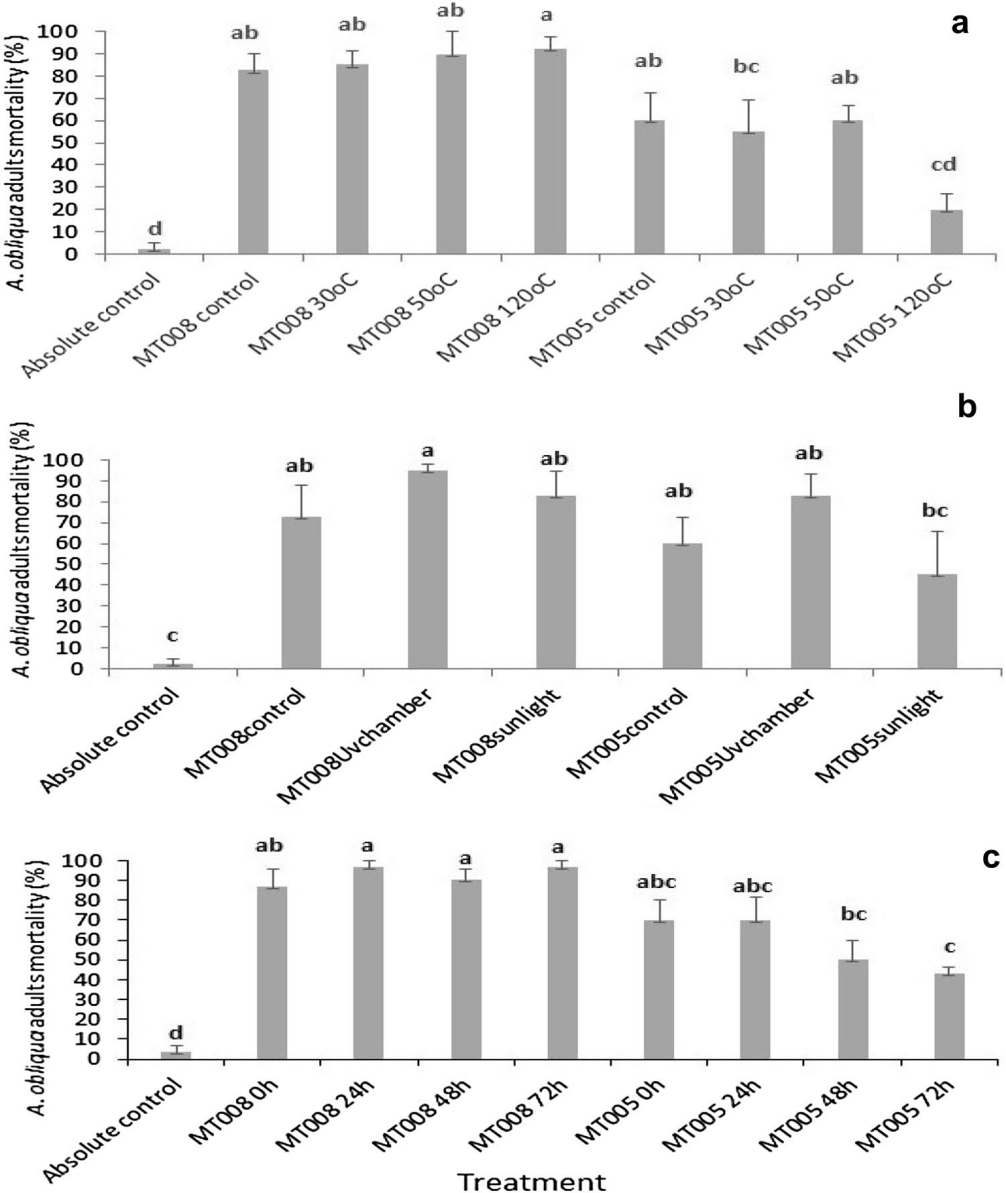
Effects of environmental factors on the insecticidal activity of crude extracts obtained from *Metarhizium robertsii* MT008 and MT005. **a** Temperature, **b** sunlight and ultraviolet light. **c** Storage under environmental conditions (30^ oC^). All the graphs show the mortality of *A. obliqua* 72 h after treatment administration (mortality % ± SE mean). Treatments with the same letter do not differ statistically. A multiple range test was run, using Tukey’s honestly significant difference (HDS 0.5%)

The effect of temperature on insecticidal activity of the crude extracts obtained from MT005 and MT008 was evaluated, and statistical differences were found (F_8,35_ = 18.20 *P* < 0.0000). The insecticidal activity of the MT008 isolate was not affected by temperature because the crude extract obtained from MT008 subjected to 30 °C ± 2 °C for 3 h, 50 °C for 3 h and 120 °C for 20 min maintained its insecticidal activity above 85%. On the other hand, the insecticidal activity of the extract obtained from MT005 was affected when it was exposed to 120 °C for 20 min, reducing its activity to 67%, with no significant differences between this treatment and the control. Furthermore, no negative effects were observed on the insecticidal activity of the extracts when these were exposed to solar radiation for four hours. Mortality of *A. obliqua* adults treated with MT008 and MT005 extracts was higher when exposed to solar radiation, with values of 95.0 ± 2.9% and 82.5 ± 11.8%, respectively. The effect of storage time of the crude extract was evaluated as well. No statistical differences among treatments were found at environmental conditions (30 °C in the shade during 0, 24, 48 and 72 h) in the MT008 extract. The insecticidal activity of the extract obtained from MT008 registered the highest mortality values ranging between 86.7 ± 8.8% and 96.7 ± 3.3% (Fig. 3).

### Fractionation and evaluation of the insecticidal activity of fractions obtained from crude extracts of *Metarhizium robertsii* MT008

**Fig. 4:**
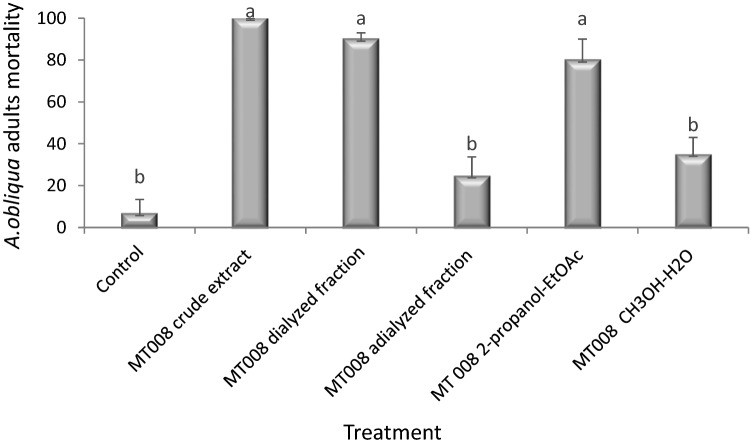
Evaluation of dialyzed, adialyzed and the organic fractions in normal phase with 2-propanol-EtOAc (8: 2 ratio) and H2O-MeOH (7:3 ratio). The graph shows the mortality of *A. obliqua* after 48 h of the treatment administration (mortality % ± SE mean). A multiple range test was run, using Tukey’s honestly significant difference (HDS 0.5%)

The evaluation of dialyzed and adialyzed fractions indicated that the insecticidal activity was retained in the dialyzed fraction, which caused a mortality 90% when evaluated; however, the adialyzed fraction showed a mortality of 24.7% (Fig. 4). Concurrently, the evaluation of organic fractions in normal phase indicated that the highest activity was retained in the fractions obtained with 2-propanol-EtOAc (8: 2 ratio), causing mortalities of 80%, after 48 h of the treatment administration (Fig. 4).

**Fig. 5:**
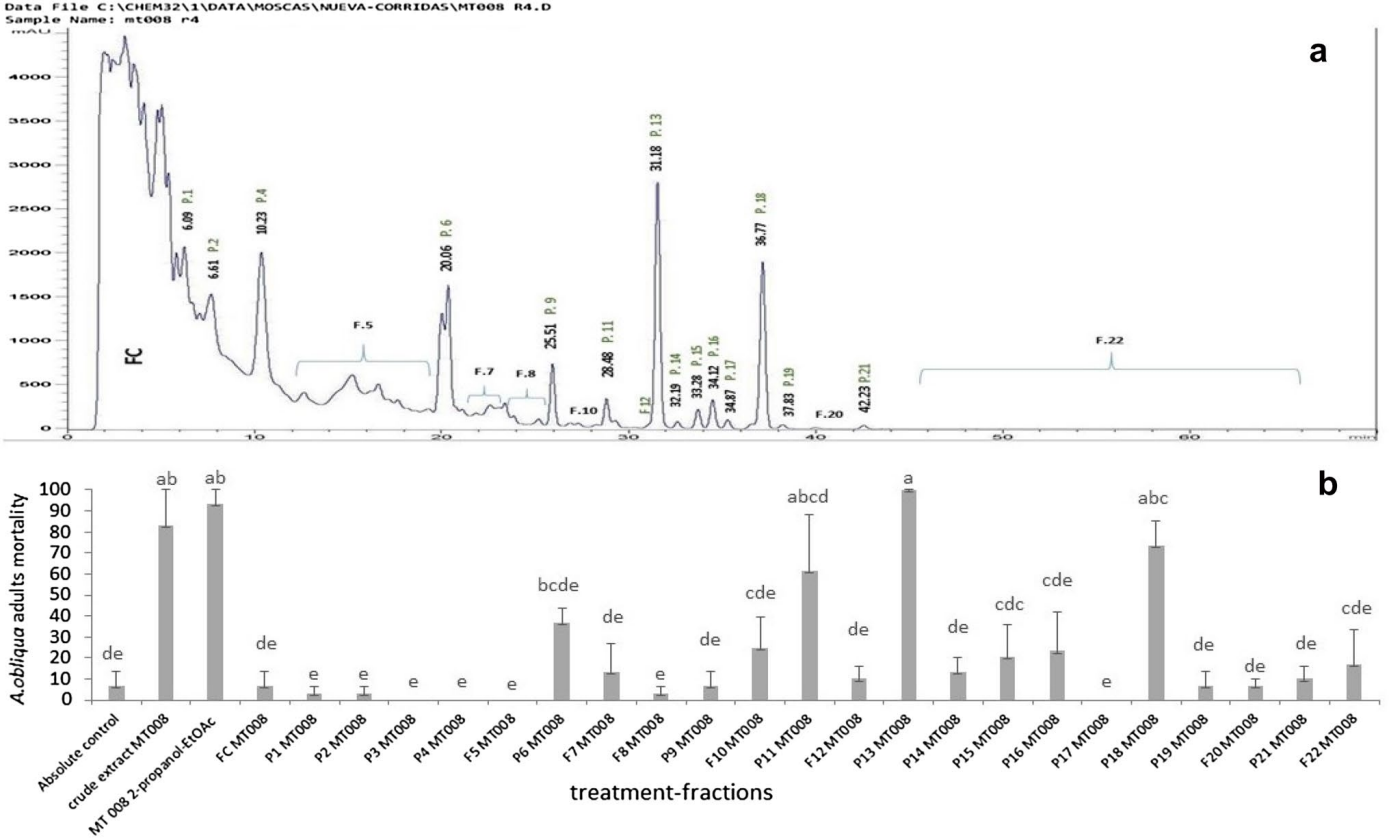
Chromatographic profile and evaluation of the HPLC fractions obtained from the extract of *M. robertsii* isolate MT008 by reverse phase on fruit fly adults, using H_2_O-CN as mobile phase: CN (80–20: 70), 1.5–2.5 ml/min and a run time of 70 min. **a** Chromatographic profile of MT008. **b** Insecticidal activity of the compounds obtained by HPLC from MT008 extract. The graph displays the average mortality of fruit fly adults on three repetitions 48 h after treatment administration. (P: defined peak, F: fraction, non-defined peak). A multiple range test was run, using Tukey’s honestly significant difference (HDS 0.5%)

HPLC fractionation of the active fraction 2-propanol-EtOAc (8: 2 ratio) obtained from the MT008 isolate extract displayed 11 defined peaks and 11 fractions. The evaluation of the insecticidal capacity of the organic fractions obtained by HPLC indicated that the extract obtained from the isolate MT008 had a compound with high activity in *A. obliqua* adults (P13, destruxin A) causing massive mortality of up to 100%, after 48 h of the treatment administration. Furthermore, two other compounds with medium activity were found (P11, destruxin A2 and P18, destruxin B), showing mortalities between 60.0 and 81.3% after 48 h (Fig. 5A and B).

### Identification of the compounds obtained from the MT008 chromatographic profile

**Fig. 6:**
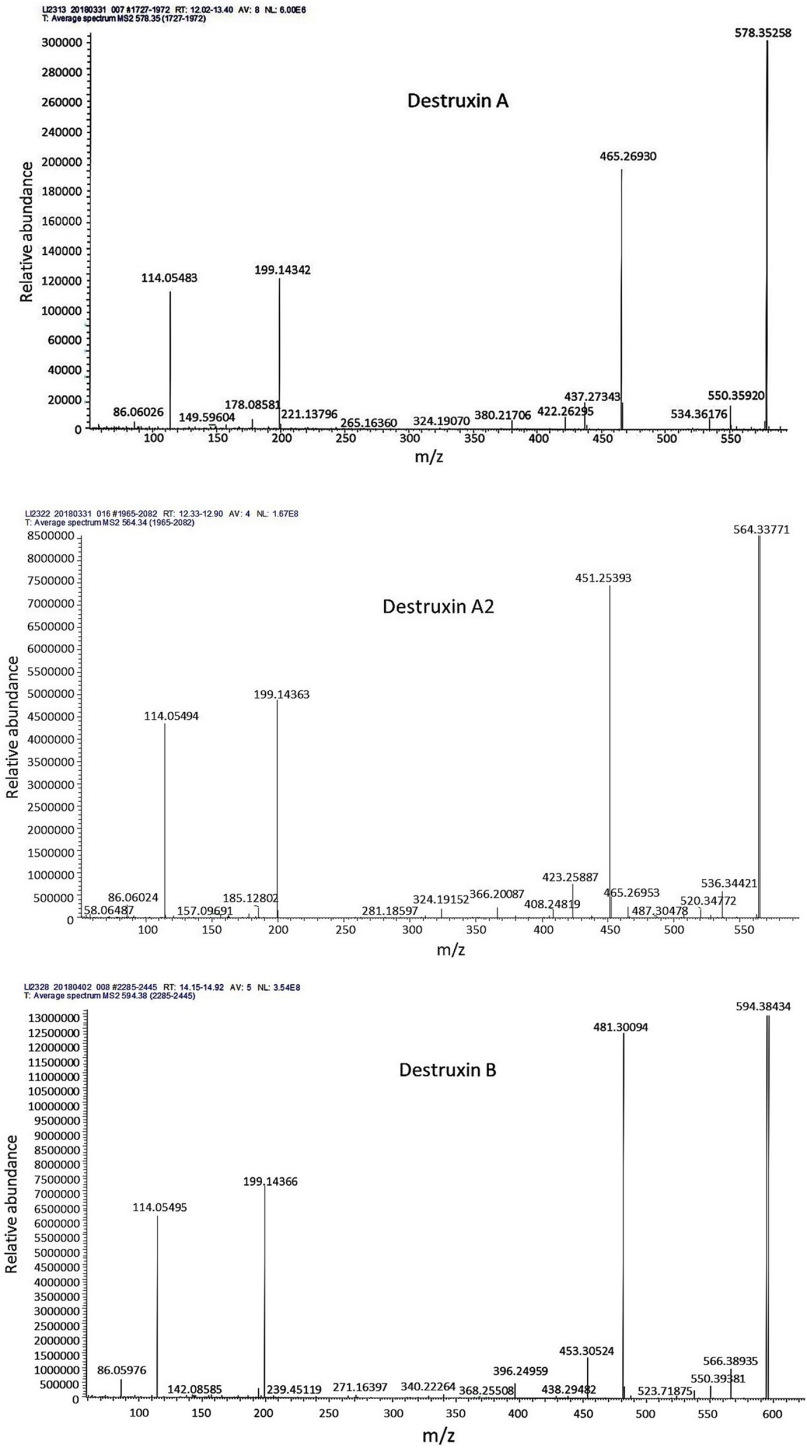
Mass/mass spectra profiles of active destruxins (A, A2 and B), obtained with several collision energies (10, 30, 50, 70 and 100) for the hydrogen adduct of the peaks analyzed

From the chromatographic profile of the MT008 extract, 12 destruxins belonging to groups E, D, A and B were determined (Table 3). The mass/mass spectra obtained with collision energies identified these as destruxin E-diol, destruxin D, destruxin D1, destruxin D2, destruxin A2, destruxin A, destruxin A3, dihydrodestruxin A, desmB, destruxin B2, destruxin B and destruxin B1. The mass/mass spectra of active destruxin (A, A2 and B) are shown in Fig. 6.

**Table 3:** Destruxins found in the extract obtained from *Metarhizium robertsii* (MT008)

Peak	Molecular mass		Fragmentation ions	Molecular formula	Compound name
	[M + H]^+^	[M + Na]^+^			
P6	612.35715	634.33850	594, 499, 471	C29H49N5O9	Destruxin E-diol
	624.35767	646.33905	511, 483, 370	C30H49N5O9	Destruxin D
	638.37347	660.35492	525, 497, 384	C31H51N5O9	Destruxin D1
P9	610.37823	632.35962	592, 497, 469	C30H51N508	Destruxin D2
P11	564.33710	586.31848	451, 423, 324	C28H45N5O7	Destruxin A2
P13	578.35223	600.33374	550, 465, 437	C29H47N5O7	Destruxin A
P14	566.35303	588.33441	467,439,340	C28H47N5O7	Destruxin A3
P15	580.36841	602.34967	552, 467, 439	C29H49N5O7	Dihydrodestruxin A
P16	580.36835	602.34973	552, 467, 439	C29H49N5O7	DesmB
P17	580.36792	602.34930	552,467,439, 368	C29H49N5O7	Destruxin B2
P18	594.38318	616.36383	566, 481, 453	C30H51N5O7	Destruxin B
P19	608.39996	630.38177	495,467	C31H53N5O7	Destruxin B1

## Discussion

*Metarhizium* species are well-known entomopathogens for their role in biological pest control. For the development of biopesticides based on these species, it is necessary to have a correct identification of the isolates. Phylogenetic studies with molecular tools have revealed the existence of closely related species in the *Metarhizium* group, corresponding to cryptic species of *M. anisopliae* (Mayerhofer et al. 2019). Four species are known as part of the clade PARB (*M. pingshaens, M. anisopliae*, *M. robertsii* and *M. brunneum*) (Rehner & Kepler 2017; Mayerhofer et al. 2019), but it is not possible to distinguish between this species using the marker (ITS). Consequently, several assignments of *Metarhizium* species as biocontrol agents (BCA) have required correction (Mayerhofer et al. 2019). In this work, using concatenated markers EF-1alpha and beta-tubulin made it possible to identify the isolates Mt005 and Mt008 as *M. robertsii*.

Of all the extracts evaluated, the one from the isolate MT008 showed high effect as insecticidal compound. Therefore, this strain, characterized as *M. robertsii*, is a potential source for generation bio-products for the control of adults of *A. obliqua* in an efficient way, due to the rapid action of its extract and its tolerance to stress factors such as sunlight, temperature and storage time. The concentrated extract of MT008 caused 95.5% of mortality, after 48 h of treatment. These results suggest a reduction in populations due to the rapid action of the compounds once they are ingested by the insects; these results agree with those obtained by Lozano-Tovar et al (2015), who found that crude extracts of the strain *Metarhizium brunneum* were efficient in the control of *C. capitata* under laboratory conditions, obtaining mortalities above 90%, 48 h after exposure. In addition, the species of *Metarhizium* are characterized by the production of secondary metabolites such as destruxins (Pedras et al. 2002). Some destruxins have been referenced with high insecticidal activity on fruit flies like *Ceratitis capitata* and *Bactrocera olea* (Lozano-Tovar et al. 2015; Yousef et al. 2013).

In general, the extract of the isolate MT008 showed higher insecticidal activity when it was subjected to 50 °C for 3 h and 120 °C for 20 min. According to Skrobek et al (2008), the temperature is a factor that strongly influences the decomposition of some destruxins, particularly dtxE. Lozano-Tovar et al (2015) showed that destruxin A2 is susceptible to high temperatures; meanwhile, the insecticidal activity of destruxin A is maintained above 90% at 120 °C for 20 min. Yousef et al (2014) showed thermostability of the insecticidal activity of extracts obtained from a *Metarhizium brunneum* when this extract was exposed to 100 °C for 3 h. In this work, solar exposure increased the concentration of the extract obtained from MT008 due to the effect of evaporation, without an adverse impact on its effectiveness as insecticide. The extract of MT005, however, showed a negative effect due to the sensitivity of its compounds at a high temperature. On the other hand, the effect of ultraviolet light on insecticidal compounds depends on its chemical structure, exposure time and wavelength (Soliman 2012). The insecticidal activity of the extracts in this study was not affected by direct solar exposure. The insecticidal activity is influenced by the exposure time as this increase the concentration of the compounds, thus increasing mortality. In general, the insecticidal properties of the crude extracts were maintained during the 72 h of storage under normal environmental conditions at 30 °C ± 2 °C. It is important to highlight this result, since this indicate that these compounds maintain their insecticidal activity in the field for a period longer than 72 h.

To conclude, chromatographic profile HPLC fractions revealed 22 compounds; twelve of these compounds were identified as destruxins as follows: destruxin E-diol, destruxin D, destruxin D1, destruxin D2, destruxin A2, destruxin A, destruxin A3, dihydrodestruxin A, desmB destruxin, destruxin B2, destruxin B and destruxin B1. Three of these destruxins (destruxin A, destruxin A2 and destruxin B) showed insecticidal activity against *A. obliqua* 48 h after ingestion, with mortalities of 100%, 60% and 81.3%, respectively. Destruxins have a strong insecticidal activity against a wide range of insects that are considered pests. The toxicity of this group of metabolites is attributed mainly to its activity of forming ionophoric and lipophilic complexes with the cell membrane of insects, compromising its structural integrity. Furthermore, as part of the mechanism of mycosis in insects, destruxins destroy the mitochondria, inhibit fluid secretion of the Malpighi tubes and cancel the immune response in the hemolymph, (Mustafa and Kaur 2013). Insect susceptibility to destruxins is variable, but in general, dtxs A and E seem to be more toxic to *Galleria* larvae than dtxs D and B (Vey et al. 2001). Similarly, studies have shown that larvae of *Musca domestica* (Diptera: Muscidae) are more susceptible to dtx E than to dtxs A or B (Vey et al. 2001), and research on adults of the Mediterranean fly (*C. capitata*) indicated that this insect is susceptible to dtxs A and A2 produced by *M. brunneum* (Lozano Tovar et al. 2015).

Taking these results into account obtained of this research, it can be said that the extract of the isolate MT008 of *M. robertsii* is a possible resource for the control of *A. obliqua.*

## Data Availability

The authors declare that the data supporting the findings of this study are available within the paper and its supporting information files. Raw data is available from the corresponding author upon reasonable request.

## Abbreviation

dtx: Destruxin
